# Characterization of hybrid cobalt-porous silicon systems: protective effect of the Matrix in the metal oxidation

**DOI:** 10.1186/1556-276X-7-495

**Published:** 2012-09-02

**Authors:** Álvaro Muñoz-Noval, Darío Gallach, Miguel Ángel García, Vicente Ferro-Llanos, Pilar Herrero, Kazuhiro Fukami, Yukio H Ogata, Vicente Torres-Costa, Raúl J Martín-Palma, Aurelio Ciment-Font, Miguel Manso-Silván

**Affiliations:** 1 Departamento de Física Aplicada, Universidad Autónoma de Madrid, Cantoblanco, Madrid 28049, Spain; 2 Departamento de Electrocerámica, Instituto de Cerámica y Vidrio (ICV-CSIC), Cantoblanco, Madrid, 28049, Spain; 3 IMDEA Nanociencia, Cantoblanco, Madrid, 28049, Spain; 4 GTB, ETSIT Telecomunicaciones, Universidad Politécnica de Madrid, Madrid, 28040, Spain; 5 Instituto de Ciencia de Materiales de Madrid (ICMM-CSIC), Cantoblanco, Madrid, 28049, Spain; 6 Institute of Advanced Energy, Kyoto University, Uji, Kyoto, 611-0011, Japan

**Keywords:** Porous silicon, Hybrid materials, Metal electroinfiltration, Transmission electron microscopy

## Abstract

In the present work, the characterization of cobalt-porous silicon (Co-PSi) hybrid systems is performed by a combination of magnetic, spectroscopic, and structural techniques. The Co-PSi structures are composed by a columnar matrix of PSi with Co nanoparticles embedded inside, as determined by Transmission Electron Microscopy (TEM). The oxidation state, crystalline structure, and magnetic behavior are determined by X-Ray Absorption Spectroscopy (XAS) and Alternating Gradient Field Magnetometry (AGFM). Additionally, the Co concentration profile inside the matrix has been studied by Rutherford Backscattering Spectroscopy (RBS). It is concluded that the PSi matrix can be tailored to provide the Co nanoparticles with extra protection against oxidation.

## Background

The development of hybrid materials is a current topic of research with many potential applications in several fields, including optoelectronics, catalysis, and biomedicine. Concretely, the hybridization of semiconductors with ferromagnetic material such as cobalt, iron, and nickel gives the possibility to obtain materials that combine semiconducting and magnetic properties [1]. Moreover, the continuous progress in nanotechnology during the last decades has led to a large availability of techniques for the fabrication and characterization of nanometric structures with controlled composition and dimensions, resulting in nanostructures with very specific properties and several functionalities [2]. In fact, multifunctional metal-based nanostructures have received a great deal of attention during the past few years given their special properties and potential applications in many scientific and technologic fields, including biomedicine [3]. In this sense, the conjugation of magnetic-semiconductor hybrid nanosystems has allowed manipulation of local spin in spintronics [4,5] and the fabrication of high sensitive magnetic sensors [6]. Regarding porous semiconductors such as porous silicon (PSi), they present additional advantages including high surface area and high surface reactivity [7].

This work aims at studying the oxidation state and crystalline structure of cobalt nanoparticles (NPs) embedded into porous silicon, resulting in Co-PSi hybrid structures. Co has been infiltrated into the PSi matrix by electrochemical techniques [8]. The suitability of PSi to host Co NPs grown by electroinfiltration has been evaluated, and both the magnetic and structural properties of the hybrid structures have been studied. The role of the porous matrix protecting Co against oxidation has been evaluated by infiltrating the Co NPs into PSi layers with different morphologies. The chemical and structural states of the Co NPs have been determined by combining highly selective and sensitive characterization techniques such as X-Ray Absorption Spectroscopy-synchrotron (XAS-synchrotron) and Rutherford Backscattering (RBS).

## Methods

### Preparation of porous silicon

**Figure 1:**
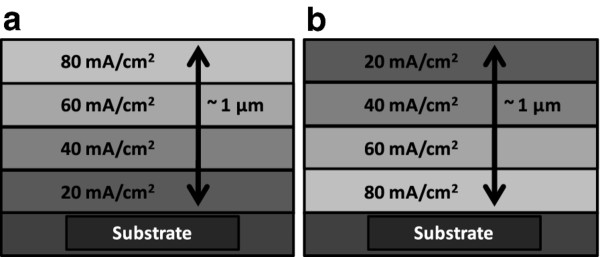
Scheme of the configuration of the PSi multilayers used for XAS experiments: (a) negative gradient configuration, Co-PSi-n; (b) positive gradient configuration, Co-PSi-p.

Porous silicon (PSi), both as single layers and multilayers, was obtained from high conductivity (0.01-0.02 Ωcm), p-type, silicon wafers. The anodization in 1:2 (volume) HF:ethanol solutions (from commercial HF 48% (w/v) in water, Sigma-Aldrich) was carried out in a homemade Teflon® electrochemical cell, with a Pt reference electrode. The Si wafers were galvanostatically etched under illumination from a 100 W halogen lamp. Single layer PSi were obtained at 100 mA/cm^2^ for 100 s, resulting in layers of about 10 microns. Different current densities were set for obtain different porosities from 40 to 120 mA/cm^2^ in case of multilayer configurations (Figure 1). Two different porosity gradients were chosen to electrochemically grow Co NPs into PSi: A negative gradient (more porous towards the surface) and a positive gradient (more porous towards the substrate). In both cases the current densities applied were in four steps: 20, 40, 60 and 80 mA/cm^2^. After etching, the Si/PSi substrates were rinsed in ethanol and dried with N_2_.

### Electroinfiltration of Co

**Figure 2:**
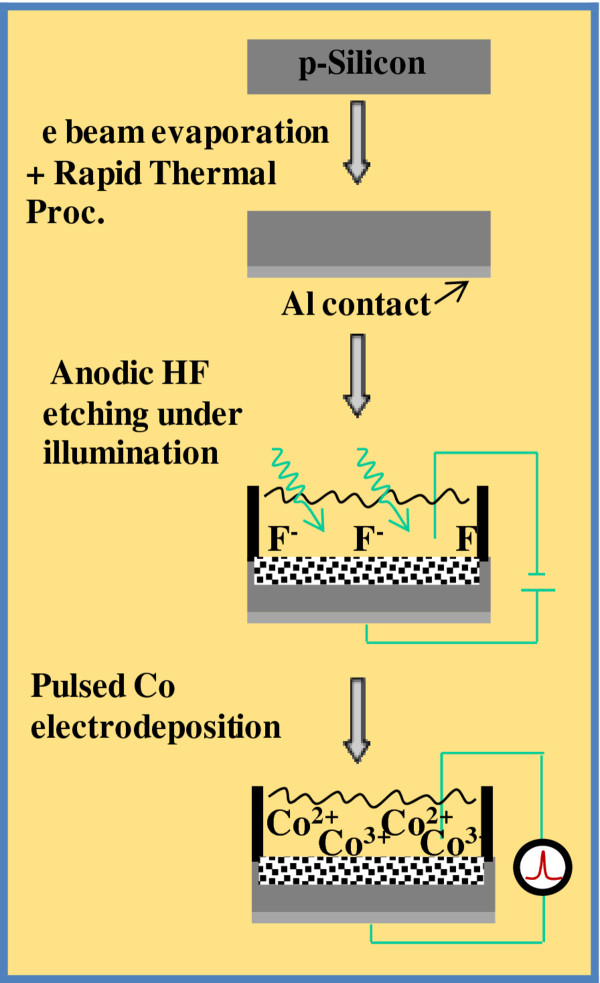
**PSi is obtained by electrochemical anodization of highly conductive p-type Si wafers in HF:ethanol solutions (1:2).** The nucleation of Co inside the matrix is achieved by electroinfiltration of Co in the porous matrix by a pulsed mode.

Electroinfiltration of Co nanoparticles into PSi single- and multi-layers was carried out in a electrochemical bath with a Watt’s solution at RT (CoSO_4_·7H_2_O 0.2 M, CoCl_2_ 0.05, H_3_BO_3_ 0.4 M, Na-Saccharine 25 g/l H_2_SO_4_ 1 mM). Boric acid was used as a buffer, to avoid pH fluctuations, sodic saccharine as crystallization catalyzer and sulfuric acid to decrease the pH of the solutions below 3. A well parameterized pulsed current process was used to allow the nucleation of metal nanoparticles into the pores of pulses of 40 mA/cm^2^ and 10 s. An important parameter in pulsed mode is the equilibrium time or rest time, between pulses, set at 60 s; estimated by observing the dynamics of the voltage versus time curves (not shown). A overall view of the whole process of PSi single/multi layer fabrication and Co electroinfiltration is summarized in Figure 2.

### Characterization

**Field emission scanning electron microscopy (FESEM)** images were obtained in a XL 30S-FEG (PHILIPS). No metallization was required to observe the samples. Samples for cross section observation were prepared according to previously optimized protocols for mechanical and ion bean milling [9]. TEM/STEM characterization was carried out using a Jeol JEM 3000F with HAADF (High Angle Annular Dark Field) system included (300kV).

#### X-ray Absorption Spectroscopy (XAS)

X-Ray Absorption Near-Edge Structure (XANES) and Extended X-ray Absorption Fine Structure (EXAFS) spectroscopy measurements at the Co K-edge energy (7704.9 eV) were performed at room temperature in fluorescence mode at the BM25 Spanish CRG Beamline (SpLine) of the ESRF (European Synchrotron Radiation Facility). An INCA 13 elements X- Ray detector was used. Bulk metallic Co, plus CoO and Co_3_O_4_ powders were measured as references. Data were normalized applying the same normalization parameters for all the spectra by means of Athena Software [10].

#### Alternating gradient field magnetometry

Magnetic characterization was performed using a Micromag Model 2900 Alternating Gradient Magnetometer System (Princeton Measurements Corporation). Nine measurements were taken for each sample. Measurements were carried out applying a magnetic field from -100mT to 100mT, a time pass of 100 ms, and a field pass of 800μT.

#### Rutherford backscatering spectroscopy

Micro analytical techniques were used to obtain in-depth elemental information. A Cockcroft-Walton tandem accelerator located at Centro de Micro-Análisis de Materiales (CMAM, Universidad Autónoma de Madrid, Spain) was used for Rutherford Backscattering Spectroscopy (RBS). RBS was performed with a 3.050 MeV He^+^ beam (incidence angle was 75° with respect to the surface normal). RBS was acquired by using silicon surface barrier detectors placed at scattering angle of 170°. A 13 μm thick mylar foil was placed in front of the detector on the forward scattering angle to stop the He forward scattered particles and filter the H recoils. All RBS experiments were performed in vacuum (pressure lower than 10E-5 mbar). All spectra were simulated using the SIMNRA code [11] to obtain the element in-depth composition.

## Results and discussion

### PSi single layers

**Figure 3:**
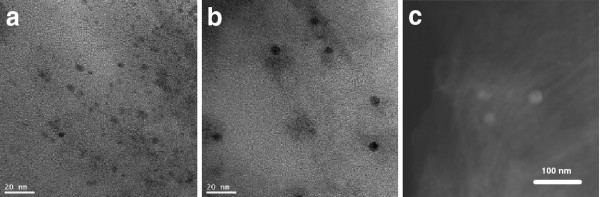
**(a,b)TEMimages of typical Co-PSi structures showing Co NPs into the PSi matrix; (c) STEM image of typical Co-PSi structures, showing the Co NPs in a brighter tone than the matrix, composed by a lighter element (Si and O).**

Preliminary structural studies of Co-PSi hybrid structures are carried out by Transmission Electron Microscopy (TEM) in a milled sample of single layer Co-PSi. As Figure 3 shows, Co ions infiltrate inside the PSi matrix and form spherical nanoparticles (NP) into the pores. In this case, nanoparticles of circa 5 nm are formed. Scanning TEM (STEM) images plus EDX analysis of these Systems (Figure 3) allow the identification of both elements in the NP.

**Figure 4:**
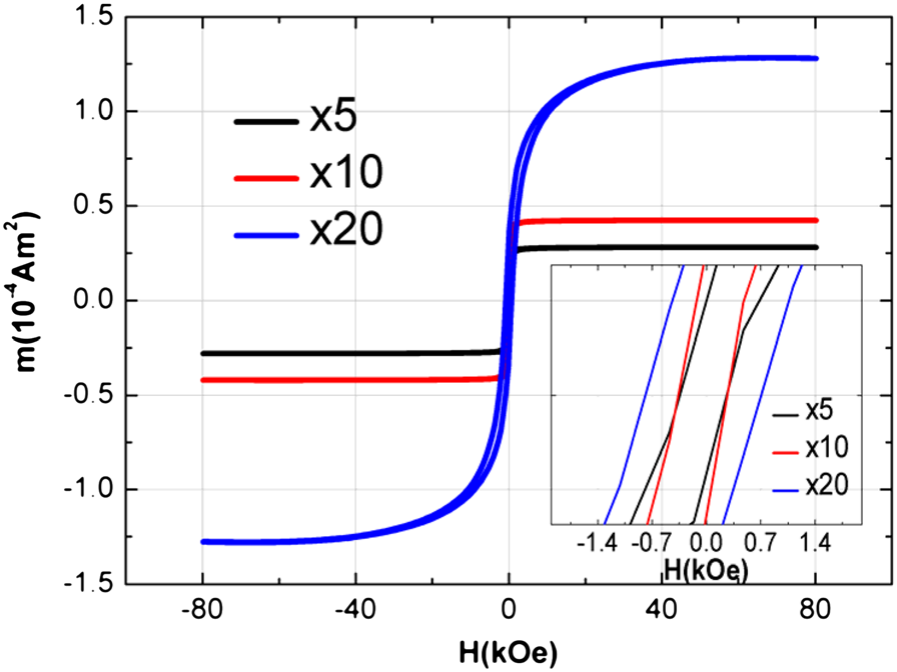
**AGFM magnetization curve for Co-PSi systems with increasing Co concentration inside the matrix, obtained from increasing number of electroinfiltration pulses: 5, 10 and 20.** Demonstrating the control of the matrix filling with Co. (inset) Axis detail showing coercivity dependence of the magnetic material for increasing amount of internalized Co nanoparticles.

Magnetic properties of single layer PSi-Co are evaluated by Alternating Gradient Field Magnetometry (AGFM). Samples electroinfiltrated at an increasing number of cycles with Co, from 5 to 20 cycles, are evaluated. Saturation magnetization values normalized to mass increase with increasing number of cycles in the studied range of cycles. Results point to the possibility of tailoring the magnetization of Co-PSi by controlling the amount of Co inside the PSi matrix (Figure 4). This is achieved by setting the number of electroinfiltration pulses during the synthesis process. Coercivity magnitude of the material depends on the amount of Co in the hybrid material: less concentrated systems present lower coercivity values [4,6]; systems with higher Co concentration present higher values. This feature can be due to the appearance of inter-particle correlations inside the matrix or by the increase of the size of particles [12] favoured by an increasing density of NPs into the PSi matrix.

### PSi multilayers

**Figure 5:**
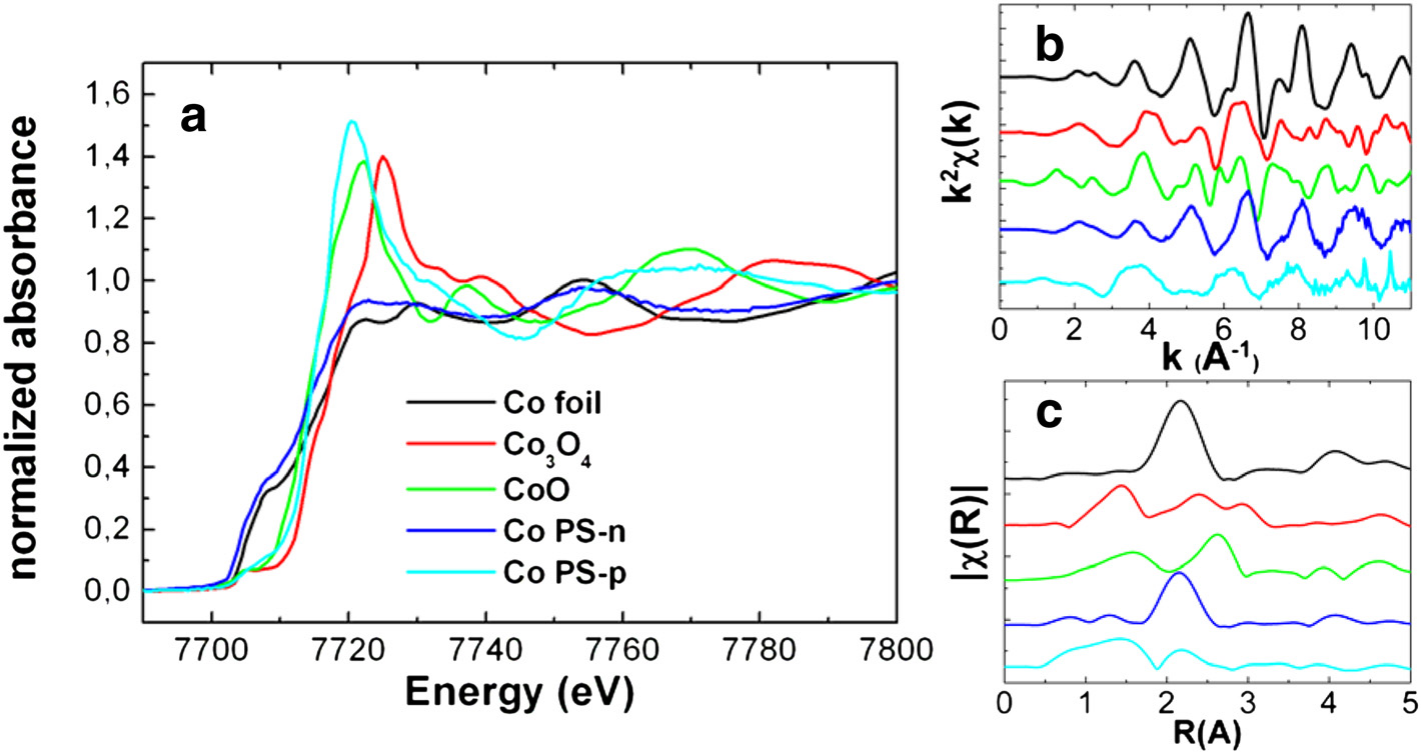
XAS spectra of the Co references and Co-PSi multilayer systems: a) XANES spectra, b) EXAFS spectra of the references and samples; c) Radial Distribution Functions of the referred samples.

Once studied the magnetic potential of Co-PSi systems, the protective effect of the PSi matrix against the oxidation of Co is determined. Magnetic properties of Co strongly depend on the oxidation state and therefore determine the possible applications of these hybrid systems [13]. For this purpose a multilayer PSi is fabricated and infiltrated with Co. Two multilayer configurations are made, as Figure 1 shows, one with a negative porosity gradient (Co-PSi-n) and other with a positive one (Co-PSi-p). These systems are studied by XAS [14-16] (Figure 5). XANES spectra (Figure 5a) of both systems show clear differences in oxidation state. Co and Cobalt oxides references are obtained for comparing and adjusting the experimental spectra to known materials. From the fitting of the experimental data to references it can be observed that in Co-PSi-n systems, Co atoms are mainly in metallic state (close to 90% at.) and the remaining 10% at. is in the form Co_3_O_4_. Besides, studying Co-PSi-p systems shows Co to be composed by a mix of Co (40%at.), CoO (50%at.) and Co_3_O_4_ (10%at). The EXAFS spectra of the Co-PSi show the element environment and lattice structure in the Fourier space (Figure 5b). Comparing the EXAFS spectra of the Co-PSi systems with the reference it is straightforward to identify similarities of that of Co-PSi-n with the spectrum of metallic Co. The similarities are clearer if we obtain the transform the spectra to the real space by obtaining the Radial Distribution Function (RDF, Figure 5c). In this figure the spacial distribution of neighbours around the scattered element is represented and gives an overall situation of the short range environment of the Co. In Figure 5c the similarities of CoPSi-n with metallic Co are clearer. In case of CoPSi-p, EXAFS spectra has more common features with CoO than with Co_3_O_4_: position and shape of the RDF in the first neighbour matches with the O in the CoO, but the second one matches with the position of Co atoms in the metallic Co, confirming the observations in XANES spectrum.

**Figure 6:**
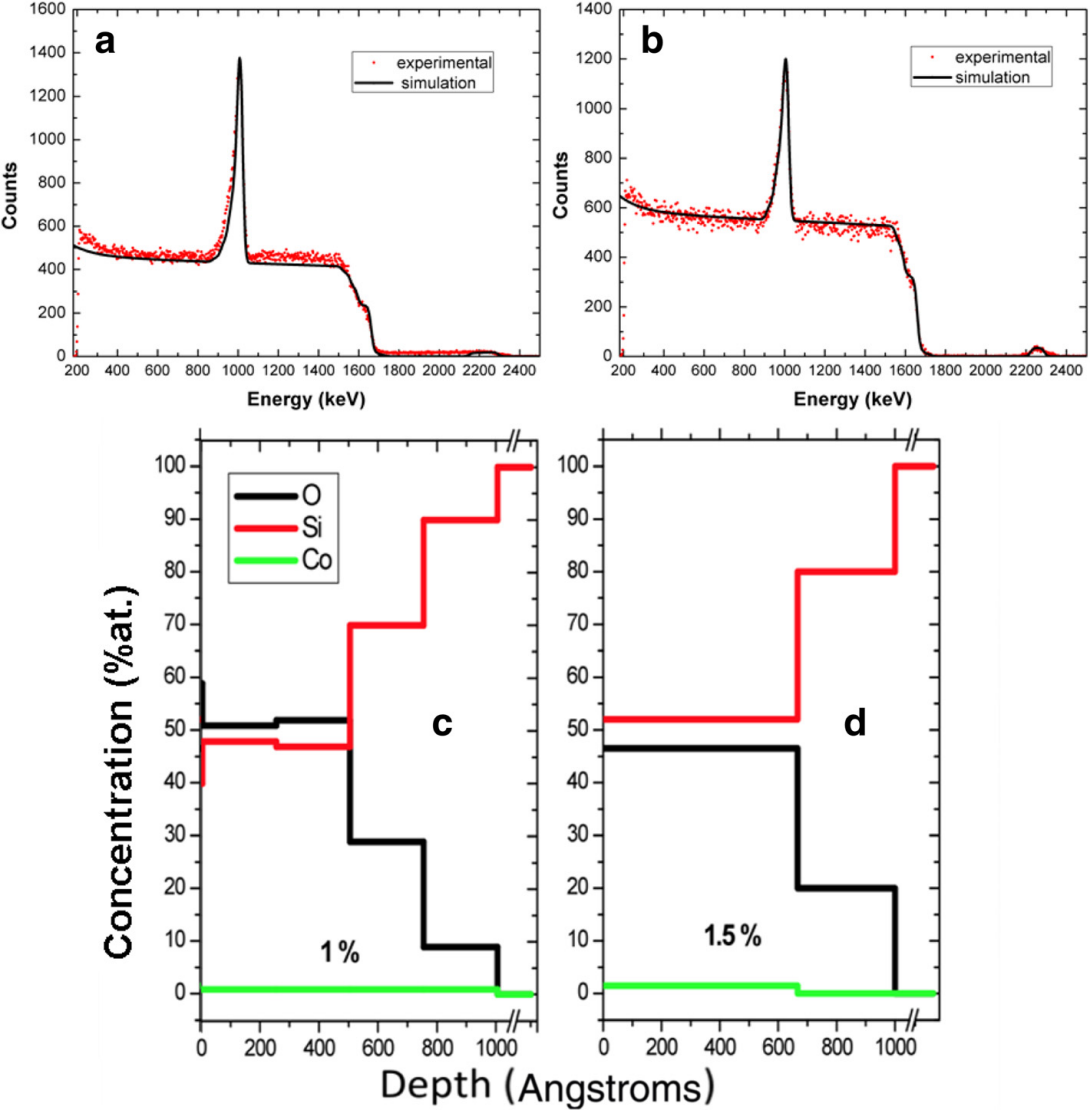
**(a,b) RBS spectra of Co-PSi-n and Co-PSi-p systems, respectively showing experimental data (dots), and simulation (lines). (c** and **d)** Elemental concentration profiles of Co-PSi-n and Co-PSi-p, respectively obtained from simulations of the spectra shown in a and b.

The elemental concentration profile of these systems is studied by Rutherford Back Scattering (RBS). RBS spectra and elemental profiles of both systems are depicted in Figure 6. Figure 6a,b, show the raw RBS spectra of the CoPSi-n and CoPSi-p samples, respectively, and the simulated spectra with the SIMNRA software [11]. A reference of Ta-Ti alternated multilayers was used to perform the energy calibration of the experimental system. By using this reference, a fine adjustment of the set up parameters was achieved. In the analyzed PSi systems, the porosity degree does not contribute significantly to the measured oxygen amount. The void (or porous) part of the samples does not produce backscattering nor energy loss, and a porous material is indistinguishable of a bulk one by this technique. This is a reason to not be able to determine porosity profiles directly by RBS. In this respect, other authors have carried out these calculations by filling pores with hydrocarbon solvents like pentane to obtain porosity profiles in PSi multilayers [17,18]. In these cases the filling substance can be quantified and an indirect measurement of the porosity can be obtained.

From the raw spectra (Figure 6a,b) we observe a clear difference in the profiles. The feature in the higher energies region corresponding to the Co presents a localized broad peak in case of Co-PSi-p and an extended region for Co-PSi-n. In a first approximation there should exists differences in the Co distribution along the layer in both configurations. Both spectra present the Si (1600–1700 keV) and O (~1000 keV) edges with subtle differences that require finer calculations. By using the fitting software it has been determined that in Co-PSi-n structures, Co is uniformly distributed along the multilayer, and the oxygen concentrates mainly near the surface. This means that metallic Co is hosted in deeper zones of the multilayer and Co_3_O_4_ is concentrated in outer zones of the matrix. For Co-PSi-p systems, Co penetrates to less deep zones and the O concentration keeps fairly constant along the multilayer. This is due both to the presence of O bubbles inside the matrix and the coexistence of a mixture of Co oxides. Figure 6c,d summarize the elemental in-depth profiles of each systems, CoPSi-n and CoPSi-p, respectively, obtained from the simulations of the experimental spectra.

**Figure 7:**
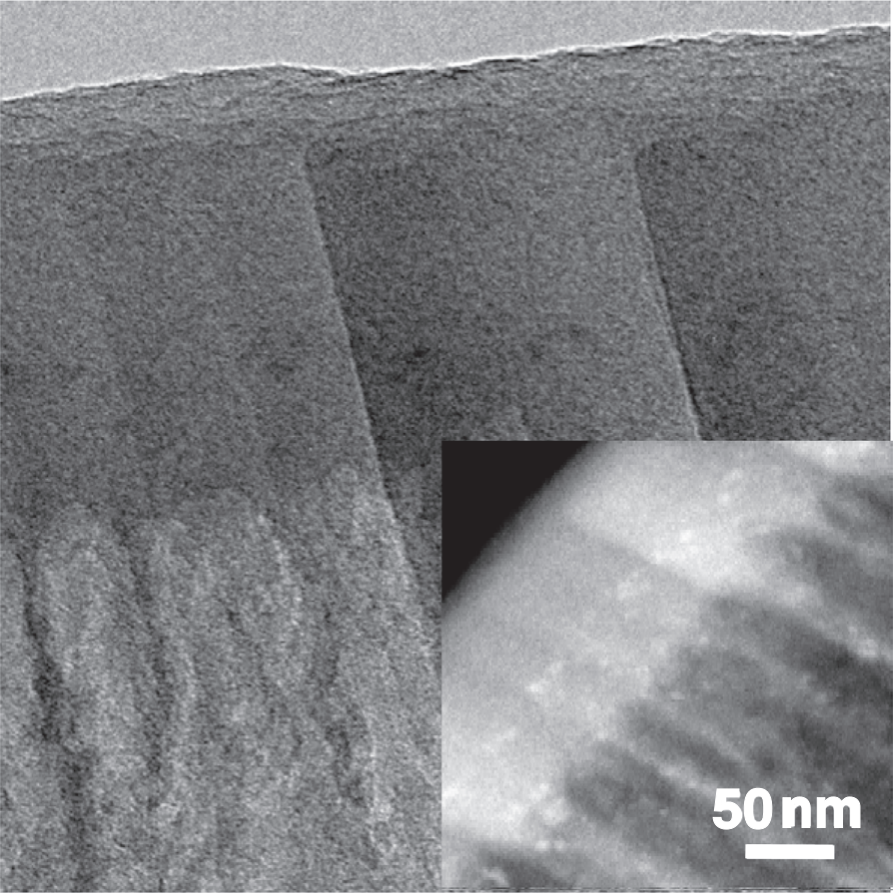
**Cross section TEM images of a sample Co-PSi-n showing the Co NPs inside the porous matrix.** The inset is a magnification in STEM mode highlighting the atomic weight of the elements.

A typical cross section of a CoPSi-n system is observed by TEM to study the distribution of the Co deposits along the PSi structure. A representative image is presented in Figure 7. In this image, the distribution of the Co NPs inside the PSi matrix can be clearly identified and also the porous structure of the PSi layer. An image in the STEM mode (inset to Figure 7), which enhances the contrast with the differences in atomic weight, facilitates the identification of Co inside the Si matrix. Again, Co NPs of circa 5 nm and spherical shape are observed inside the PSi matrix, with a roughly uniform distribution of them inside the porous structure.

## Conclusions

In this work the protective effect of the matrix on the oxidation of electroinfiltrated Co nanoparticles into PSi has been proved. Moreover, a selected multilayer configuration in the matrix allows minimizing the formation of Co oxides that forms an oxidation profile in the whole layer depending on the porosity gradient of the PSi template. The magnetic properties of such Co-PSi systems suggest the possibility to control the magnetization of these hybrid materials by controlling the amount of infiltrated Co. Nevertheless, the influence of the PSi porosity type and degree in the oxidation of the hosted Co need to be further characterized. Future work in this sense should be performed in order to identify the physico-chemical properties of the PSi matrix that minimizes the oxidation in such hybrid materials.
